# Complete mitochondrial genome of the Devil Ray, *Mobula thurstoni* (Lloyd, 1908) (Myliobatiformes: Myliobatidae)

**DOI:** 10.1080/23802359.2017.1407689

**Published:** 2017-11-26

**Authors:** Betsaida Santillán-Lugo, Raúl Llera-Herrera, David Corro-Espinosa, Erick C. Oñate-González, Guillermo Rodríguez-Domínguez, Nancy C. Saavedra-Sotelo

**Affiliations:** a Facultad de Ciencias del Mar, Universidad Autónoma de Sinaloa, Mazatlán, Mexico;; b Unidad en Acuicultura y Manejo Ambiental, CONACyT-Centro de Investigación en Alimentación y Desarrollo A. C., Mazatlán, Mexico;; c Centro Regional de investigaciones Pesqueras de Mazatlán, Instituto Nacional de Pesca, Mazatlán, Mexico;; d Instituto de Ciencias del Mar y Limnología, Unidad Academica Mazatlan, Universidad Nacional Autónoma de México, Mazatlán, Mexico;; e Facultad de Ciencias del Mar, CONACYT-Universidad Autónoma de Sinaloa, Mazatlán, Mexico

**Keywords:** Ray, *Mobula*, mitogenome, Illumina

## Abstract

The Devil Ray (*Mobula thurstoni*) is a species with global distribution and is an important species in conservation terms, here we present its complete mitochondrial genome assembled with Illumina sequencing data. The circular genome was 17,610 bp in length, and consists of 13 protein-coding, two ribosomal RNAs (rRNAs), and 22 transfer RNA (tRNA) genes. Base composition is 30.7% A, 29.1% T, 26.5% C, and 13.7% G, and 40.2% GC content. Protein-coding genes present two start codon (ATG and GUG) and seven stop codon (UAA, AUA, UUU, UUA, AAU, CCU, and UAG). The control region possesses the highest A + T (66.6%) content among all mitochondrial regions. These data would contribute to the evolutionary studies of this genus, where there has been recent reclassification.

The Devil Ray, *Mobula thurstoni*, is a large ray with a reported circumglobal distribution in tropical and temperate waters but in scattered localities (Eschmeyer et al. 1983; Last and Stevens 1994; Compagno and Last 1999; Compagno 1999; Walls et al. 2016). In general, *M. thurstoni* is a large ray with 180 cm of disc width (DW), has a colouration of dark-blue to black dorsally and white colour ventrally, and pectoral fin tip of silvery colour (Notarbartolo-di-Sciara 1987). It is a pelagic species that forms shoals in coastal and oceanic waters, presents sexual segregation and low productivity (one pup each year), which makes susceptible to fishing (Notarbartolo-di-Sciara 1987; Compagno 1999; Serrano-López 2009; Couturier et al. 2012). Due to this, some conservation efforts have been established; at the international level it was included in the IUCN Red List classified as Near Threatened (Walls et al. 2016) and was included in Appendix II of CITES since 2016. Particularly to México, there is a fishing ban for this (and other) species, which was established in the NOM-029-PESC-2006 (DOF. Diario Oficial de la Federación 2007).

To determine the complete mitogenome of *M. thurstoni*, we collected three samples of skin tissue from specimens captured and released after the biopsies in the locality of La Reforma, Sinaloa, Mexico (25°04′17.6″N 108°36′49.4″W). Genomic DNA was extracted using the Wizard^®^ Genomic DNA Purification kit (Promega, Madison, WI), which was stored in the Molecular Ecology Laboratory of the Universidad Autónoma de Sinaloa (23°12′31″N 106°25′35″W). A genomic DNA library was constructed with the kapa gDNA library kit (Kapa Biosystems, Wilmington, MA) using multiplex index. The library was sequenced alongside other barcoded libraries using a single lane (2 × 125 paired-end reads) in the MiSeq platform (Illumina, San Diego, CA). The reads resulting from sequencing were pre-processed using PRINSEQ lite (Schmieder and Edwards 2011) to remove residual adapters, and Trimmomatic v0.33 (Bolger et al. 2014) for trim low-quality ends (*Q* score <20), and remove reads shorter than 80 bases. Sequences were demultiplexed, and the recovered reads were analysed for quality control with FastQC v0.10.1 (Babraham Institute, Cambridge, UK) (Andrews 2010). 11,381,140 pair of high-quality reads (*Q* score >25) were recovered. Genome partial assembling in silico with shorts reads was done using MITObim v1.7 (Hahn et al. 2013) with the mitogenome of the Japanese Devil Ray *Mobula japanica* as a reference (GenBank accession: JX392983.1). In addition, the gaps into assembled mitogenome were filled using Sanger sequencing, in which six paired primers were designed. Final assembly was annotated using MitoAnnotator and Mitofish (Iwasaki et al. 2013).

The complete mitogenome of *M. thurstoni* has a length of 17,610 bp (GenBank accession number MG206065) and a base composition of A 30.7%, T 29.1%, C 26.5%, and G 13.7%, and the GC content of 40.2%. The mitogenome contains all typical genes found in most vertebrate mitogenomes: 13 protein coding genes, 22 transference RNA genes, two ribosomal RNAs (rRNAs), and one control region or d-loop. Protein coding genes initiate by the typical AUG codon, except for the *COX1* gene, which presented GUG as start codon; rest of genes present seven stop codons (UAA, AUA, UUU, UUA, AAU, CCU, and UAG). *D-loop* region was 1884 bp, presenting the highest A + T content of 66.6% among all mitochondrial regions.

We validated the phylogenetic position of *M. thurstoni* with a maximum-likelihood tree (500 bootstrap replicates) of complete and partial mtDNA from the other 12 species of rays using MEGA6 (Tamura et al. 2013). The phylogenetic position o*f M. thurstoni* was close to all species of Myliobatidae family (Figure 1). A recent study showed that *Manta* genus disappear and its two species pass to *Mobula* genus (White et al. 2017). In addition, the mitochondrial topology showed to *M. japanica* and *M. mobular* were the same evolutionary independent unit as previously described in others phylogenetic analyses; the same happens with *M. eregoodootenkee* and *M. kuhlii* (Poortvliet et al. 2015; Bustamante et al. 2016; White et al. 2017).

**Figure 1.**
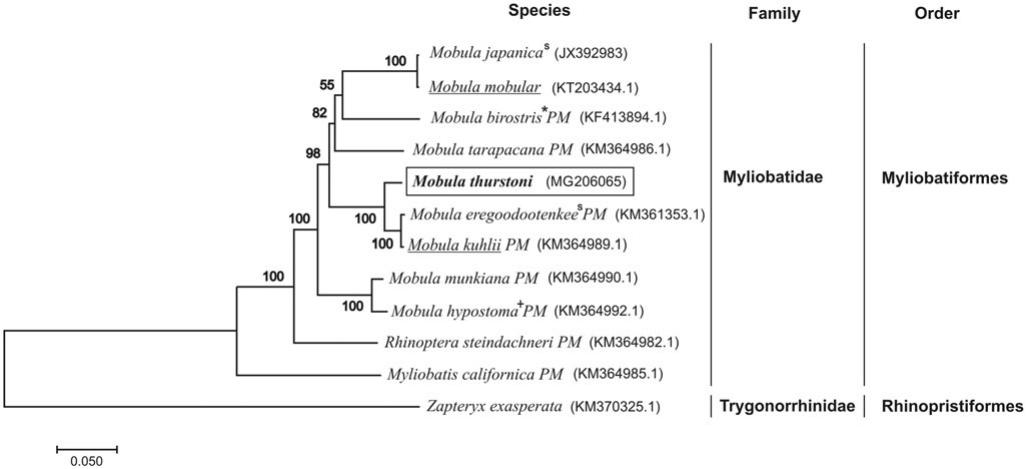
Maximum-likelihood (ML) phylogenetic tree of *Mobula thurstoni* (rectangle) and the other eight species of Myliobatidae family, using *Zapteryx exasperata* as outgroup. Symbols and abbreviations: partial mitogenome (PM); before *Manta birostris* (*); before *Mobula rochebrunei* (+); synonyms (^s^) of *Mobula mobular* and *Mobula kuhlii* respectively (underlined). Number above each node indicates the ML bootstrap support values. In parenthesis, the access numbers from NCBI database.
